# New distributional data on ascidian fauna (Tunicata: Ascidiacea) from Mandapam coast, Gulf of Mannar, India

**DOI:** 10.3897/BDJ.4.e7855

**Published:** 2016-03-02

**Authors:** Abdul Jaffarali, Soban A Akram, Kaleem ML Arshan

**Affiliations:** ‡ Islamiah College (Autonomous), Vaniyambadi, India

**Keywords:** Ascidian, diversity, Gulf of Mannar, India, Mandapam.

## Abstract

**Background:**

Ascidians play a key role in the ecology and biodiversity of marine ecosystem. Ascidians can be transported in ship ballast water and while attached to ship and boat hulls. Heavy traffic by domestic and international ships as well as cargo vessels between the major and minor ports warrants continuous monitoring for new introductions of ascidians. The Mandapam coast is situated in the Gulf of Mannar, India, a marine hot spot area in the Indian Ocean which provides an environment suitable for the settlement of ascidians.

**New information:**

A total of 30 species of ascidians were reported from Mandapam coastal waters, of which 26 species were new to the study area and five species: *Ecteinascidia turbinata*, *Eudistoma carnosum*, *Trididemnum caelatum*, *T. vermiforme* and *Didemnum spadix*, were new to India.

## Introduction

Ascidians (Class: Ascidiacea and subphylum: Urochordata), commonly called tunicates, are a common, diverse group among the macro fouling communities in marine ecosystems, that attach to natural and artificial substrates in the intertidal and subtidal zones of coastal habitats throughout the world. They are one of the key ecological groups because of their invasive potential. Ascidians have the potential to produce new lead molecules with significant pharmacological activities (Davidson 1993; Rinehart 2000; Haefner 2003 and Jain et al. 2008). Some ascidians have been used for their nutritional value as food for people in many parts of the world including India (Nanri et al. 1992 and Tamilselvi et al. 2010). Certain solitary ascidians serve as indicators of water quality (Abdul Jaffar Ali 2004; Tamilselvi 2008; Abdul Jaffar Ali et al. 2011 and Abdul Jaffar Ali et al. 2015b).

India has a rich natural heritage and nurtures a unique biodiversity, placing it among the 17 most biodiverse countries (Venkataraman 2003). The Gulf of Mannar, located in the southeast coast of India, is a hot spot for Indian marine biodiversity and recognized as Marine National Park. The site provides natural and artificial substrata for the successive settlement, growth and breeding of ascidian species throughout the year.

Mandapam (Latitude 9°28’N and Longitude 79°15’E) is located in Gulf of Mannar and experiences heavy traffic of fishing and defense vessels from various major and minor ports along the Indian coast. Tidal exchange in the area is seasonal. Direction and force of the monsoonal winds influence this station, since the coast line in the east-west direction.

## Materials and methods

The study was carried out from March 2014 to February 2015 at Mandapam, India. Collections were made randomly during the study period at intertidal beds and in shallow water regions. Hand tools were used to remove animals from solid surfaces like pillars of jetties, small rocks and hulls of fishing vessels. Snorkeling was also employed to collect ascidians at 1-2 meter depths. Collected specimens were narcotized with menthol and then preserved in 10% buffered formalin in seawater. The specimens were sorted and identified to species or the lowest possible taxonomic level using taxonomic keys (Sebastian 1956; Tokioka 1967; Millar 1975; Kott 1985; Renganathan 1986; Monniot and Monniot 1996 and Kott 2001). Data set of distribution of ascidians throughout the world was obtained from WoRMs.

## Taxon treatments

### Ecteinascidia thurstoni

Herdman, 1906

http://eol.org/pages/513367/overview

#### Materials

- **Type status:** Other material. **Occurrence:** catalogNumber: DBTICMPM01; recordedBy: Abdul Jaffarali et al.; individualCount: 2; sex: Hermophrodite; lifeStage: adult; **Taxon:** kingdom: Animalia; phylum: Tunicata; class: Ascidiacea; order: Phlebobranchia; family: Perophoridae; genus: Ecteinascidia; specificEpithet: thurstoni; scientificNameAuthorship: Herdman, 1890; **Location:** continent: Asia; country: India; stateProvince: Tamil Nadu; municipality: Ramanathapuram; locality: Mandapam; locationRemarks: Intertidal flats and shallow water; decimalLatitude: 9.2856; decimalLongitude: 79.1586; **Identification:** identifiedBy: Dr. H. Abdul Jaffar Ali; dateIdentified: 2015; **Event:** samplingProtocol: Hand picking; year: 2015; month: 2; day: 9; eventRemarks: H. Abdul Jaffarali, A. Soban Akram, M.L. Kaleem Arshan; **Record Level:** type: Physical Object; language: en; institutionID: IC; collectionID: MPM/PB/04; collectionCode: Ascidians; basisOfRecord: PreservedSpecimen

#### Distribution

Andaman Sea, Mediterranean Sea - Eastern Basin, Thailand, Djibouti, South Pacific Ocean

##### Distribution in India

Gulf of Mannar.

### Ecteinascidia turbinata

Herdman, 1880

http://eol.org/pages/513369/overview

#### Materials

- **Type status:** Other material. **Occurrence:** catalogNumber: DBTICMPM02; recordedBy: Abdul Jaffarali et al.; individualCount: 2; sex: Hermophrodite; lifeStage: adult; **Taxon:** kingdom: Animalia; phylum: Tunicata; class: Ascidiacea; order: Phlebobranchia; family: Perophoridae; genus: Ecteinascidia; specificEpithet: turbinata; scientificNameAuthorship: Herdman, 1880; **Location:** continent: Asia; country: India; stateProvince: Tamil Nadu; municipality: Ramanathapuram; locality: Mandapam; locationRemarks: Intertidal flats and shallow water; decimalLatitude: 9.2856; decimalLongitude: 79.1586; **Identification:** identifiedBy: Dr. H. Abdul Jaffar Ali; dateIdentified: 2015; **Event:** samplingProtocol: Hand picking; year: 2015; month: 2; day: 9; eventRemarks: H. Abdul Jaffarali, A. Soban Akram, M.L. Kaleem Arshan; **Record Level:** type: Physical Object; language: en; institutionID: IC; collectionID: MPM/PB/04; collectionCode: Ascidians; basisOfRecord: PreservedSpecimen

#### Distribution

United States, Djibouti, Gulf of Mexico, Mediterranean Sea, North Atlantic Ocean.

##### Distribution in India

Mandapam.

### Ecteinascidia venui

Meenakshi, 1997

http://eol.org/pages/513370/overview

#### Materials

- **Type status:** Other material. **Occurrence:** catalogNumber: DBTICMPM03; recordedBy: Abdul Jaffarali et al.; individualCount: 2; sex: Hermophrodite; lifeStage: adult; **Taxon:** taxonID: Native; kingdom: Animalia; phylum: Tunicata; class: Ascidiacea; order: Phlebobranchia; family: Perophoridae; genus: Ecteinascidia; specificEpithet: venui; scientificNameAuthorship: Meenakshi, 1997; **Location:** continent: Asia; country: India; stateProvince: Tamil Nadu; municipality: Ramanathapuram; locality: Mandapam; locationRemarks: Intertidal flats and shallow water; decimalLatitude: 9.2856; decimalLongitude: 79.1586; **Identification:** identifiedBy: Dr. H. Abdul Jaffar Ali; dateIdentified: 2014; **Event:** samplingProtocol: Hand picking; year: 2014; month: 8; day: 27; eventRemarks: H. Abdul Jaffarali, A. Soban Akram, M.L. Kaleem Arshan; **Record Level:** type: Physical Object; language: en; institutionID: IC; collectionID: MPM/PB/03; collectionCode: Ascidians; basisOfRecord: PreservedSpecimen

#### Distribution

India

##### Distribution in India

Thoothukudi coast.

### Symplegma oceania

Tokioka, 1961

http://eol.org/pages/515533/overview

#### Materials

- **Type status:** Other material. **Occurrence:** catalogNumber: DBTICMPM04; recordedBy: Abdul Jaffarali et al.; individualCount: 1; sex: Hermophrodite; lifeStage: adult; **Taxon:** taxonID: Cryptogenic; kingdom: Animalia; phylum: Tunicata; class: Ascidiacea; order: Stolidobranchia; family: Styelidae; genus: Symplegma; specificEpithet: oceania; scientificNameAuthorship: Tokioka, 1961; **Location:** continent: Asia; country: India; stateProvince: Tamil Nadu; municipality: Ramanathapuram; locality: Mandapam; locationRemarks: Intertidal flats and shallow water; decimalLatitude: 9.2856; decimalLongitude: 79.1586; **Identification:** identifiedBy: Dr. H. Abdul Jaffar Ali; dateIdentified: 2014; **Event:** samplingProtocol: Peeling off; year: 2014; month: 3; day: 15; eventRemarks: H. Abdul Jaffarali, A. Soban Akram, M.L. Kaleem Arshan; **Record Level:** type: Physical Object; language: en; institutionID: IC; collectionID: MPM/PB/01; collectionCode: Ascidians; basisOfRecord: PreservedSpecimen

#### Distribution

Indonesia, Hong Kong, China (People's Republic), Palau, New Caledonia, Fiji, Sri Lanka, Northern Territory (N coast), Queensland (Central East coast, Great Barrier Reef, Northeast coast), South Australia (Great Australian Bight, South Gulfs coast), Victoria (Bass Strait), Western Australia (Central West coast, Lower West coast, Northwest coast); Noumea, also West Indian Ocean, Gulf of Mexico, Caribbean.

##### Distribution in India

Thoothukudi coast, Leepuram, China Muttom, Colachel & Vizhinjam Bay.

### Styela canopus

(Savigny, 1816)

http://eol.org/pages/503604/overview

#### Materials

- **Type status:** Other material. **Occurrence:** catalogNumber: DBTICMPM05; recordedBy: Abdul Jaffarali et al.; individualCount: 2; sex: Hermophrodite; lifeStage: adult; **Taxon:** taxonID: Invasive; kingdom: Animalia; phylum: Tunicata; class: Ascidiacea; order: Stolidobranchia; family: Styelidae; genus: Styela; specificEpithet: canopus; scientificNameAuthorship: (Savigny, 1816); **Location:** continent: Asia; country: India; stateProvince: Tamil Nadu; municipality: Ramanathapuram; locality: Mandapam; locationRemarks: Intertidal flats and shallow water; decimalLatitude: 9.2856; decimalLongitude: 79.1586; **Identification:** identifiedBy: Dr. H. Abdul Jaffar Ali; dateIdentified: 2014; **Event:** samplingProtocol: Dislodging; year: 2014; month: 3; day: 15; eventRemarks: H. Abdul Jaffarali, A. Soban Akram, M.L. Kaleem Arshan; **Record Level:** type: Physical Object; language: en; institutionID: IC; collectionID: MPM/PB/01; collectionCode: Ascidians; basisOfRecord: PreservedSpecimen

#### Distribution

Japan, France, Indonesia, Hong Kong, New South Wales (Central East coast), Queensland (Central East coast, Northeast coast), Western Australia (Lower West coast, Northwest coast); Torres Strait, Coral Sea, west Pacific Ocean, Korea, tropical and temperate Atlantic Ocean, Persian Gulf, Adriatic, Mediterranean, Ascension Is., Channel Is., west coast of France.

##### Distribution in India

Thoothukudi coast and Vizhinjam Bay.

### Microcosmus exasperatus

Heller, 1878

http://eol.org/pages/514239/overview

#### Materials

- **Type status:** Other material. **Occurrence:** catalogNumber: DBTICMPM06; recordedBy: Abdul Jaffarali et al.; individualCount: 1; sex: Hermophrodite; lifeStage: adult; **Taxon:** taxonID: Invasive; kingdom: Animalia; phylum: Tunicata; class: Ascidiacea; order: Stolidobranchia; family: Pyuridae; genus: Microcosmus; specificEpithet: exasperatus; scientificNameAuthorship: Heller, 1878; **Location:** continent: Asia; country: India; stateProvince: Tamil Nadu; municipality: Ramanathapuram; locality: Mandapam; locationRemarks: Intertidal flats and shallow water; decimalLatitude: 9.2856; decimalLongitude: 79.1586; **Identification:** identifiedBy: Dr. H. Abdul Jaffar Ali; dateIdentified: 2014; **Event:** samplingProtocol: Dislodging; year: 2014; month: 3; day: 15; eventRemarks: H. Abdul Jaffarali, A. Soban Akram, M.L. Kaleem Arshan; **Record Level:** type: Physical Object; language: en; institutionID: IC; collectionID: MPM/PB/01; collectionCode: Ascidians; basisOfRecord: PreservedSpecimen

#### Distribution

Fiji, Philippines, Hawaii, New Caledonia, Bermuda, Brazil, Indonesia, Florida, New South Wales (Central East coast, Lower East coast), Northern Territory (North coast), Queensland (Central East coast, Northeast coast), Western Australia (Central West coast, Lower West coast, North coast, Northwest coast); West Indies, East Africa, Red Sea.

##### Distribution in India

Thoothukudi coast, Chinna Muttom and Vizhinjam Bay.

### Microcosmus propinquus

Herdman, 1882

http://eol.org/pages/514253/overview

#### Materials

- **Type status:** Other material. **Occurrence:** catalogNumber: DBTICMPM07; recordedBy: Abdul Jaffarali et al.; individualCount: 1; sex: Hermophrodite; lifeStage: adult; **Taxon:** taxonID: Cryptogenic; kingdom: Animalia; phylum: Tunicata; class: Ascidiacea; order: Stolidobranchia; family: Pyuridae; genus: Microcosmus; specificEpithet: propinquus; scientificNameAuthorship: Herdman, 1882; **Location:** continent: Asia; country: India; stateProvince: Tamil Nadu; municipality: Ramanathapuram; locality: Mandapam; locationRemarks: Intertidal flats and shallow water; decimalLatitude: 9.2856; decimalLongitude: 79.1586; **Identification:** identifiedBy: Dr. H. Abdul Jaffar Ali; dateIdentified: 2014; **Event:** samplingProtocol: Dislodging; year: 2014; month: 3; day: 15; eventRemarks: H. Abdul Jaffarali, A. Soban Akram, M.L. Kaleem Arshan; **Record Level:** type: Physical Object; language: en; institutionID: IC; collectionID: MPM/PB/01; collectionCode: Ascidians; basisOfRecord: PreservedSpecimen

#### Distribution

Australia

##### Distribution in India

Vizhinjam Bay.

### Eudistoma carnosum

Kott, 1990

http://eol.org/pages/513439/overview

#### Materials

- **Type status:** Other material. **Occurrence:** catalogNumber: DBTICMPM08; recordedBy: Abdul Jaffarali et al.; individualCount: 1; sex: Hermophrodite; lifeStage: adult; **Taxon:** kingdom: Animalia; phylum: Tunicata; class: Ascidiacea; order: Aplousobranchia; family: Polycitoridae; genus: Eudistoma; specificEpithet: carnosum; scientificNameAuthorship: Kott, 1990; **Location:** continent: Asia; country: India; stateProvince: Tamil Nadu; municipality: Ramanathapuram; locality: Mandapam; locationRemarks: Intertidal flats and shallow water; decimalLatitude: 9.2856; decimalLongitude: 79.1586; **Identification:** identifiedBy: Dr. H. Abdul Jaffar Ali; dateIdentified: 2014; **Event:** samplingProtocol: Hand picking; year: 2014; month: 3; day: 15; eventRemarks: H. Abdul Jaffarali, A. Soban Akram, M.L. Kaleem Arshan; **Record Level:** type: Physical Object; language: en; institutionID: IC; collectionID: MPM/PB/01; collectionCode: Ascidians; basisOfRecord: PreservedSpecimen

#### Distribution

Australia

##### Distribution in India

Mandapam.

### Eudistoma gilboviride

(Sluiter, 1909)

http://eol.org/pages/513450/overview

#### Materials

- **Type status:** Other material. **Occurrence:** catalogNumber: DBTICMPM09; recordedBy: Abdul Jaffarali et al.; individualCount: 1; sex: Hermophrodite; lifeStage: adult; **Taxon:** kingdom: Animalia; phylum: Tunicata; class: Ascidiacea; order: Aplousobranchia; family: Polycitoridae; genus: Eudistoma; specificEpithet: gilboviride; scientificNameAuthorship: (Sluiter, 1909); **Location:** continent: Asia; country: India; stateProvince: Tamil Nadu; municipality: Ramanathapuram; locality: Mandapam; locationRemarks: Intertidal flats and shallow water; decimalLatitude: 9.2856; decimalLongitude: 79.1586; **Identification:** identifiedBy: Dr. H. Abdul Jaffar Ali; dateIdentified: 2014; **Event:** samplingProtocol: Hand picking; year: 2014; month: 8; day: 27; eventRemarks: H. Abdul Jaffarali, A. Soban Akram, M.L. Kaleem Arshan; **Record Level:** type: Physical Object; language: en; institutionID: IC; collectionID: MPM/PB/03; collectionCode: Ascidians; basisOfRecord: PreservedSpecimen

#### Distribution

Central Indo-Pacific.

##### Distribution in India

Gulf of Mannar.

### Eudistoma pyriforme

(Herdman, 1886)

http://eol.org/pages/513493/overview

#### Materials

- **Type status:** Other material. **Occurrence:** catalogNumber: DBTICMPM10; recordedBy: Abdul Jaffarali et al.; individualCount: 3; sex: Hermophrodite; lifeStage: adult; **Taxon:** taxonID: Invasive; kingdom: Animalia; phylum: Tunicata; class: Ascidiacea; order: Aplousobranchia; family: Polycitoridae; genus: Eudistoma; specificEpithet: pyriforme; scientificNameAuthorship: (Herdman, 1886); **Location:** continent: Asia; country: India; stateProvince: Tamil Nadu; municipality: Ramanathapuram; locality: Mandapam; locationRemarks: Intertidal flats and shallow water; decimalLatitude: 9.2856; decimalLongitude: 79.1586; **Identification:** identifiedBy: Dr. H. Abdul Jaffar Ali; dateIdentified: 2014; **Event:** samplingProtocol: Hand picking; year: 2014; month: 3; day: 15; eventRemarks: H. Abdul Jaffarali, A. Soban Akram, M.L. Kaleem Arshan; **Record Level:** type: Physical Object; language: en; institutionID: IC; collectionID: MPM/PB/01; collectionCode: Ascidians; basisOfRecord: PreservedSpecimen

#### Distribution

Madagascar, Central Indo-Pacific.

##### Distribution in India

Tiruchendur.

### Eudistoma viride

Tokioka, 1955

http://eol.org/pages/513512/overview

#### Materials

- **Type status:** Other material. **Occurrence:** catalogNumber: DBTICMPM11; recordedBy: Abdul Jaffarali et al.; individualCount: 5; sex: Hermophrodite; lifeStage: adult; **Taxon:** taxonID: Established Invasive; kingdom: Animalia; phylum: Tunicata; class: Ascidiacea; order: Aplousobranchia; family: Polycitoridae; genus: Eudistoma; specificEpithet: viride; scientificNameAuthorship: Tokioka, 1955; **Location:** continent: Asia; country: India; stateProvince: Tamil Nadu; municipality: Ramanathapuram; locality: Mandapam; locationRemarks: Intertidal flats and shallow water; decimalLatitude: 9.2856; decimalLongitude: 79.1586; **Identification:** identifiedBy: Dr. H. Abdul Jaffar Ali; dateIdentified: 2015; **Event:** samplingProtocol: Hand picking; year: 2015; month: 2; day: 9; eventRemarks: H. Abdul Jaffarali, A. Soban Akram, M.L. Kaleem Arshan; **Record Level:** type: Physical Object; language: en; institutionID: IC; collectionID: MPM/PB/04; collectionCode: Ascidians; basisOfRecord: PreservedSpecimen

#### Distribution

Central Indo-Pacific.

##### Distribution in India

Thoothukudi coast.

### Synoicum galei

Kott, 1992

http://eol.org/pages/513842/overview

#### Materials

- **Type status:** Other material. **Occurrence:** catalogNumber: DBTICMPM12; recordedBy: Abdul Jaffarali et al.; individualCount: 3; sex: Hermophrodite; lifeStage: adult; **Taxon:** kingdom: Animalia; phylum: Tunicata; class: Ascidiacea; order: Aplousobranchia; family: Polyclinidae; genus: Synoicum; specificEpithet: galei; scientificNameAuthorship: Kott, 1992; **Location:** continent: Asia; country: India; stateProvince: Tamil Nadu; municipality: Ramanathapuram; locality: Mandapam; locationRemarks: Intertidal flats and shallow water; decimalLatitude: 9.2856; decimalLongitude: 79.1586; **Identification:** identifiedBy: Dr. H. Abdul Jaffar Ali; dateIdentified: 2014; **Event:** samplingProtocol: Hand picking; year: 2014; month: 5; day: 12; eventRemarks: H. Abdul Jaffarali, A. Soban Akram, M.L. Kaleem Arshan; **Record Level:** type: Physical Object; language: en; institutionID: IC; collectionID: MPM/PB/02; collectionCode: Ascidians; basisOfRecord: PreservedSpecimen

#### Distribution

Australia

##### Distribution in India

Mandapam.

### Polyclinum glabrum

Sluiter, 1895

http://eol.org/pages/513775/overview

#### Materials

- **Type status:** Other material. **Occurrence:** catalogNumber: DBTICMPM13; recordedBy: Abdul Jaffarali et al.; individualCount: 6; sex: Hermophrodite; lifeStage: adult; **Taxon:** taxonID: Invasive; kingdom: Animalia; phylum: Tunicata; class: Ascidiacea; order: Aplousobranchia; family: Polyclinidae; genus: Polyclinum; specificEpithet: glabrum; scientificNameAuthorship: Sluiter, 1895; **Location:** continent: Asia; country: India; stateProvince: Tamil Nadu; municipality: Ramanathapuram; locality: Mandapam; locationRemarks: Intertidal flats and shallow water; decimalLatitude: 9.2856; decimalLongitude: 79.1586; **Identification:** identifiedBy: Dr. H. Abdul Jaffar Ali; dateIdentified: 2014; **Event:** samplingProtocol: Hand picking; year: 2014; month: 5; day: 12; eventRemarks: H. Abdul Jaffarali, A. Soban Akram, M.L. Kaleem Arshan; **Record Level:** type: Physical Object; language: en; institutionID: IC; collectionID: MPM/PB/02; collectionCode: Ascidians; basisOfRecord: PreservedSpecimen

#### Distribution

Indonesia, Northern Territory (North coast), Queensland (Great Barrier Reef, Northeast coast), Western Australia (Northwest coast).

##### Distribution in India

Vizhinjam Bay.

### Polyclinum nudum

Kott, 1992

http://eol.org/pages/513786/overview

#### Materials

- **Type status:** Other material. **Occurrence:** catalogNumber: DBTICMPM14; recordedBy: Abdul Jaffarali et al.; individualCount: 2; sex: Hermophrodite; lifeStage: adult; **Taxon:** taxonID: Cryptogenic; kingdom: Animalia; phylum: Tunicata; class: Ascidiacea; order: Aplousobranchia; family: Polyclinidae; genus: Polyclinum; specificEpithet: nudum; scientificNameAuthorship: Kott, 1992; **Location:** continent: Asia; country: India; stateProvince: Tamil Nadu; municipality: Ramanathapuram; locality: Mandapam; locationRemarks: Intertidal flats and shallow water; decimalLatitude: 9.2856; decimalLongitude: 79.1586; **Identification:** identifiedBy: Dr. H. Abdul Jaffar Ali; dateIdentified: 2014; **Event:** samplingProtocol: Hand picking; year: 2014; month: 5; day: 12; eventRemarks: H. Abdul Jaffarali, A. Soban Akram, M.L. Kaleem Arshan; **Record Level:** type: Physical Object; language: en; institutionID: IC; collectionID: MPM/PB/02; collectionCode: Ascidians; basisOfRecord: PreservedSpecimen

#### Distribution

New South Wales (Lower East coast).

##### Distribution in India

Vizhinjam Bay.

### Polyclinum saturnium

Savigny, 1816

http://eol.org/pages/513792/overview

#### Materials

- **Type status:** Other material. **Occurrence:** catalogNumber: DBTICMPM15; recordedBy: Abdul Jaffarali et al.; individualCount: 2; sex: Hermophrodite; lifeStage: adult; **Taxon:** taxonID: Cryptogenic; kingdom: Animalia; phylum: Tunicata; class: Ascidiacea; order: Aplousobranchia; family: Polyclinidae; genus: Polyclinum; specificEpithet: saturnium; scientificNameAuthorship: Savigny, 1816; **Location:** continent: Asia; country: India; stateProvince: Tamil Nadu; municipality: Ramanathapuram; locality: Mandapam; locationRemarks: Intertidal flats and shallow water; decimalLatitude: 9.2856; decimalLongitude: 79.1586; **Identification:** identifiedBy: Dr. H. Abdul Jaffar Ali; dateIdentified: 2014; **Event:** samplingProtocol: Hand picking; year: 2014; month: 3; day: 15; eventRemarks: H. Abdul Jaffarali, A. Soban Akram, M.L. Kaleem Arshan; **Record Level:** type: Physical Object; language: en; institutionID: IC; collectionID: MPM/PB/01; collectionCode: Ascidians; basisOfRecord: PreservedSpecimen

#### Distribution

North Atlantic Ocean.

##### Distribution in India

Vizhinjam Bay.

### Polyclinum solum

Kott, 1992

http://www.gbif.org/species/5200515

#### Materials

- **Type status:** Other material. **Occurrence:** catalogNumber: DBTICMPM16; recordedBy: Abdul Jaffarali et al.; individualCount: 1; sex: Hermophrodite; lifeStage: adult; **Taxon:** taxonID: Cryptogenic; kingdom: Animalia; phylum: Tunicata; class: Ascidiacea; order: Aplousobranchia; family: Polyclinidae; genus: Polyclinum; specificEpithet: solum; scientificNameAuthorship: Kott, 1992; **Location:** continent: Asia; country: India; stateProvince: Tamil Nadu; municipality: Ramanathapuram; locality: Mandapam; locationRemarks: Intertidal flats and shallow water; decimalLatitude: 9.2856; decimalLongitude: 79.1586; **Identification:** identifiedBy: Dr. H. Abdul Jaffar Ali; dateIdentified: 2014; **Event:** samplingProtocol: Hand picking; year: 2014; month: 3; day: 15; eventRemarks: H. Abdul Jaffarali, A. Soban Akram, M.L. Kaleem Arshan; **Record Level:** type: Physical Object; language: en; institutionID: IC; collectionID: MPM/PB/01; collectionCode: Ascidians; basisOfRecord: PreservedSpecimen

#### Distribution

Australia, Indonesia.

##### Distribution in India

Gulf of Mannar.

### Polyclinum tenuatum

Kott, 1992

http://eol.org/pages/513797/overview

#### Materials

- **Type status:** Other material. **Occurrence:** catalogNumber: DBTICMPM17; recordedBy: Abdul Jaffarali et al.; individualCount: 2; sex: Hermophrodite; lifeStage: adult; **Taxon:** taxonID: Cryptogenic; kingdom: Animalia; phylum: Tunicata; class: Ascidiacea; order: Aplousobranchia; family: Polyclinidae; genus: Polyclinum; specificEpithet: tenuatum; scientificNameAuthorship: Kott, 1992; **Location:** continent: Asia; country: India; stateProvince: Tamil Nadu; municipality: Ramanathapuram; locality: Mandapam; locationRemarks: Intertidal flats and shallow water; decimalLatitude: 9.2856; decimalLongitude: 79.1586; **Identification:** identifiedBy: Dr. H. Abdul Jaffar Ali; dateIdentified: 2014; **Event:** samplingProtocol: Hand picking; year: 2014; month: 5; day: 12; eventRemarks: H. Abdul Jaffarali, A. Soban Akram, M.L. Kaleem Arshan; **Record Level:** type: Physical Object; language: en; institutionID: IC; collectionID: MPM/PB/02; collectionCode: Ascidians; basisOfRecord: PreservedSpecimen

#### Distribution

Australia.

##### Distribution in India

Chinna Muttom and Leepuram.

### Aplidiopsis confluata

Kott, 1992

http://eol.org/pages/513543/overview

#### Materials

- **Type status:** Other material. **Occurrence:** catalogNumber: DBTICMPM18; recordedBy: Abdul Jaffarali et al.; individualCount: 2; sex: Hermophrodite; lifeStage: adult; **Taxon:** kingdom: Animalia; phylum: Tunicata; class: Ascidiacea; order: Aplousobranchia; family: Polyclinidae; genus: Aplidiopsis; specificEpithet: confluata; scientificNameAuthorship: Kott, 1992; **Location:** continent: Asia; country: India; stateProvince: Tamil Nadu; municipality: Ramanathapuram; locality: Mandapam; locationRemarks: Intertidal flats and shallow water; decimalLatitude: 9.2856; decimalLongitude: 79.1586; **Identification:** identifiedBy: Dr. H. Abdul Jaffar Ali; dateIdentified: 2014; **Event:** samplingProtocol: Hand picking; year: 2014; month: 5; day: 12; eventRemarks: H. Abdul Jaffarali, A. Soban Akram, M.L. Kaleem Arshan; **Record Level:** type: Physical Object; language: en; institutionID: IC; collectionID: MPM/PB/02; collectionCode: Ascidians; basisOfRecord: PreservedSpecimen

#### Distribution

Australia

##### Distribution in India

Vizhinjam Bay.

### Trididemnum caelatum

Kott, 2001

http://eol.org/pages/513175/overview

#### Materials

- **Type status:** Other material. **Occurrence:** catalogNumber: DBTICMPM19; recordedBy: Abdul Jaffarali et al.; individualCount: 1; sex: Hermophrodite; lifeStage: adult; **Taxon:** taxonID: Cryptogenic; kingdom: Animalia; phylum: Tunicata; class: Ascidiacea; order: Aplousobranchia; family: Didemnidae; genus: Trididemnum; specificEpithet: caelatum; scientificNameAuthorship: Kott, 2001; **Location:** continent: Asia; country: India; stateProvince: Tamil Nadu; municipality: Ramanathapuram; locality: Mandapam; locationRemarks: Intertidal flats and shallow water; decimalLatitude: 9.2856; decimalLongitude: 79.1586; **Identification:** identifiedBy: Dr. H. Abdul Jaffar Ali; dateIdentified: 2014; **Event:** samplingProtocol: Peeling off; year: 2014; month: 3; day: 15; eventRemarks: H. Abdul Jaffarali, A. Soban Akram, M.L. Kaleem Arshan; **Record Level:** type: Physical Object; language: en; institutionID: IC; collectionID: MPM/PB/01; collectionCode: Ascidians; basisOfRecord: PreservedSpecimen

#### Distribution

Australia.

##### Distribution in India

Mandapam.

### Trididemnum clinides

Kott, 1977

http://eol.org/pages/513178/overview

#### Materials

- **Type status:** Other material. **Occurrence:** catalogNumber: DBTICMPM20; recordedBy: Abdul Jaffarali et al.; individualCount: 1; sex: Hermophrodite; lifeStage: adult; **Taxon:** kingdom: Animalia; phylum: Tunicata; class: Ascidiacea; order: Aplousobranchia; family: Didemnidae; genus: Trididemnum; specificEpithet: clinides; scientificNameAuthorship: Kott, 1977; **Location:** continent: Asia; country: India; stateProvince: Tamil Nadu; municipality: Ramanathapuram; locality: Mandapam; locationRemarks: Intertidal flats and shallow water; decimalLatitude: 9.2856; decimalLongitude: 79.1586; **Identification:** identifiedBy: Dr. H. Abdul Jaffar Ali; dateIdentified: 2014; **Event:** samplingProtocol: Peeling off; year: 2014; month: 3; day: 15; eventRemarks: H. Abdul Jaffarali, A. Soban Akram, M.L. Kaleem Arshan; **Record Level:** type: Physical Object; language: en; institutionID: IC; collectionID: MPM/PB/01; collectionCode: Ascidians; basisOfRecord: PreservedSpecimen

#### Distribution

South Pacific Ocean.

##### Distribution in India

Thoothukudi coast and Vizhinjam Bay.

### Trididemnum cyclops

Michaelsen, 1921

http://eol.org/pages/513180/overview

#### Materials

- **Type status:** Other material. **Occurrence:** catalogNumber: DBTICMPM21; recordedBy: Abdul Jaffarali et al.; individualCount: 1; sex: Hermophrodite; lifeStage: adult; **Taxon:** taxonID: Cryptogenic; kingdom: Animalia; phylum: Tunicata; class: Ascidiacea; order: Aplousobranchia; family: Didemnidae; genus: Trididemnum; specificEpithet: cyclops; scientificNameAuthorship: Michaelsen, 1921; **Location:** continent: Asia; country: India; stateProvince: Tamil Nadu; municipality: Ramanathapuram; locality: Mandapam; locationRemarks: Intertidal flats and shallow water; decimalLatitude: 9.2856; decimalLongitude: 79.1586; **Identification:** identifiedBy: Dr. H. Abdul Jaffar Ali; dateIdentified: 2014; **Event:** samplingProtocol: Peeling off; year: 2014; month: 3; day: 15; eventRemarks: H. Abdul Jaffarali, A. Soban Akram, M.L. Kaleem Arshan; **Record Level:** type: Physical Object; language: en; institutionID: IC; collectionID: MPM/PB/01; collectionCode: Ascidians; basisOfRecord: PreservedSpecimen

#### Distribution

South Pacific Ocean.

##### Distribution in India

Colachel.

### Trididemnum savignii

(Herdman, 1886)

http://eol.org/pages/513210/overview

#### Materials

- **Type status:** Other material. **Occurrence:** catalogNumber: DBTICMPM22; recordedBy: Abdul Jaffarali et al.; individualCount: 1; sex: Hermophrodite; lifeStage: adult; **Taxon:** taxonID: Cryptogenic; kingdom: Animalia; phylum: Tunicata; class: Ascidiacea; order: Aplousobranchia; family: Didemnidae; genus: Trididemnum; specificEpithet: savignii; scientificNameAuthorship: (Herdman, 1886); **Location:** continent: Asia; country: India; stateProvince: Tamil Nadu; municipality: Ramanathapuram; locality: Mandapam; locationRemarks: Intertidal flats and shallow water; decimalLatitude: 9.2856; decimalLongitude: 79.1586; **Identification:** identifiedBy: Dr. H. Abdul Jaffar Ali; dateIdentified: 2014; **Event:** samplingProtocol: Peeling off; year: 2014; month: 3; day: 15; eventRemarks: H. Abdul Jaffarali, A. Soban Akram, M.L. Kaleem Arshan; **Record Level:** type: Physical Object; language: en; institutionID: IC; collectionID: MPM/PB/01; collectionCode: Ascidians; basisOfRecord: PreservedSpecimen

#### Distribution

Indonesia, Bermuda, Florida, Northern Territory (North coast), Queensland (Northeast coast), Western Australia (Northwest coast); West Indies.

##### Distribution in India

Thoothukudi and Colachel.

### Trididemnum vermiforme

Kott, 2001

http://eol.org/pages/513223/overview

#### Materials

- **Type status:** Other material. **Occurrence:** catalogNumber: DBTICMPM23; recordedBy: Abdul Jaffarali et al.; individualCount: 1; sex: Hermophrodite; lifeStage: adult; **Taxon:** taxonID: Cryptogenic; kingdom: Animalia; phylum: Tunicata; class: Ascidiacea; order: Aplousobranchia; family: Didemnidae; genus: Trididemnum; specificEpithet: vermiforme; scientificNameAuthorship: Kott, 2001; **Location:** continent: Asia; country: India; stateProvince: Tamil Nadu; municipality: Ramanathapuram; locality: Mandapam; locationRemarks: Intertidal flats and shallow water; decimalLatitude: 9.2856; decimalLongitude: 79.1586; **Identification:** identifiedBy: Dr. H. Abdul Jaffar Ali; dateIdentified: 2014; **Event:** samplingProtocol: Peeling off; year: 2014; month: 3; day: 15; eventRemarks: H. Abdul Jaffarali, A. Soban Akram, M.L. Kaleem Arshan; **Record Level:** type: Physical Object; language: en; institutionID: IC; collectionID: MPM/PB/01; collectionCode: Ascidians; basisOfRecord: PreservedSpecimen

#### Distribution

Australia.

##### Distribution in India

Mandapam.

### Didemnum psammatodes

(Sluiter, 1895)

http://eol.org/pages/512886/overview

#### Materials

- **Type status:** Other material. **Occurrence:** catalogNumber: DBTICMPM24; recordedBy: Abdul Jaffarali et al.; individualCount: 4; sex: Hermophrodite; lifeStage: adult; **Taxon:** taxonID: Established Cryptogenic; kingdom: Animalia; phylum: Tunicata; class: Ascidiacea; order: Aplousobranchia; family: Didemnidae; genus: Didemnum; specificEpithet: psammathodes; scientificNameAuthorship: (Sluiter, 1895); **Location:** continent: Asia; country: India; stateProvince: Tamil Nadu; municipality: Ramanathapuram; locality: Mandapam; locationRemarks: Intertidal flats and shallow water; decimalLatitude: 9.2856; decimalLongitude: 79.1586; **Identification:** identifiedBy: Dr. H. Abdul Jaffar Ali; dateIdentified: 2015; **Event:** samplingProtocol: Peeling off; year: 2015; month: 2; day: 9; eventRemarks: H. Abdul Jaffarali, A. Soban Akram, M.L. Kaleem Arshan; **Record Level:** type: Physical Object; language: en; institutionID: IC; collectionID: MPM/PB/04; collectionCode: Ascidians; basisOfRecord: PreservedSpecimen

#### Distribution

Japan, Malaysia, Indonesia, New Zealand, New South Wales (Central East coast), Queensland (Central East coast, Great Barrier Reef, Northeast coast), Victoria (Bass Strait); west Indian Ocean, Red Sea.

##### Distribution in India

Thoothukudi coast, Leepuram, Chinna Muttom and Vizhinjam Bay.

### Didemnum spadix

Kott, 2001

http://eol.org/pages/512900/overview

#### Materials

- **Type status:** Other material. **Occurrence:** catalogNumber: DBTICMPM25; recordedBy: Abdul Jaffarali et al.; individualCount: 1; sex: Hermophrodite; lifeStage: adult; **Taxon:** kingdom: Animalia; phylum: Tunicata; class: Ascidiacea; order: Aplousobranchia; family: Didemnidae; genus: Didemnum; specificEpithet: spadix; scientificNameAuthorship: Kott, 2001; **Location:** continent: Asia; country: India; stateProvince: Tamil Nadu; municipality: Ramanathapuram; locality: Mandapam; locationRemarks: Intertidal flats and shallow water; decimalLatitude: 9.2856; decimalLongitude: 79.1586; **Identification:** identifiedBy: Dr. H. Abdul Jaffar Ali; dateIdentified: 2014; **Event:** samplingProtocol: Peeling off; year: 2014; month: 3; day: 15; eventRemarks: H. Abdul Jaffarali, A. Soban Akram, M.L. Kaleem Arshan; **Record Level:** type: Physical Object; language: en; institutionID: IC; collectionID: MPM/PB/01; collectionCode: Ascidians; basisOfRecord: PreservedSpecimen

#### Distribution

Australia.

##### Distribution in India:

Mandapam.

### Lissoclinum bistratum

Sluiter, 1905

http://eol.org/pages/513037/overview

#### Materials

- **Type status:** Other material. **Occurrence:** catalogNumber: DBTICMPM26; recordedBy: Abdul Jaffarali et al.; individualCount: 4; sex: Hermophrodite; lifeStage: adult; **Taxon:** taxonID: Invasive; kingdom: Animalia; phylum: Tunicata; class: Ascidiacea; order: Aplousobranchia; family: Didemnidae; genus: Lissoclinum; specificEpithet: bistratum; scientificNameAuthorship: Sluiter, 1905; **Location:** continent: Asia; country: India; stateProvince: Tamil Nadu; municipality: Ramanathapuram; locality: Mandapam; locationRemarks: Intertidal flats and shallow water; decimalLatitude: 9.2856; decimalLongitude: 79.1586; **Identification:** identifiedBy: Dr. H. Abdul Jaffar Ali; dateIdentified: 2015; **Event:** samplingProtocol: Peeling off; year: 2015; month: 2; day: 9; eventRemarks: H. Abdul Jaffarali, A. Soban Akram, M.L. Kaleem Arshan; **Record Level:** type: Physical Object; language: en; institutionID: IC; collectionID: MPM/PB/04; collectionCode: Ascidians; basisOfRecord: PreservedSpecimen

#### Distribution

Djibouti, South Pacific Ocean, Tanzania.

##### Distribution in India

Thoothukudi.

## Discussion

A total of 30 species of ascidians, comprising 6 families and 11 genera, from the Mandapam water has been reported in the present study (Suppl. material 1). Out of 30 species, 26 were new to this station, among which 5 species: *Ecteinascidia turbinata*, *Eudistoma carnosum*, *Trididemnum caelatum*, *T. vermiforme* and *Didemnum spadix*, were reported for the first time in India. The other 4 species were previously documented at the study area and are considered non-native species. The total number of ascidian species (n=30) is greater than previously documented (n=19) in 2003, thus increasing biodiversity. This new entry of ascidians to the study station could possibly be explained by invasiveness of ascidians and vectors such as the hulls of ships and ballast water (Byers 2002). Heavy traffic of shipping and fishing vessels acts as an important vector for intra and inter coastal spread of invaders (Johnson et al. 2001, Wasson et al. 2001, Darbyson 2009, Rocha et al. 2010).

Previously, a total of 19 ascidian species was reported from this station (Meenakshi 1997, Meenakshi 1998, Meenakshi 1999, Meenakshi 2002, Meenakshi 2003, Meenakshi 2006, Meenakshi 2009, Renganathan 1984a, Renganathan 1984b), among which only four species (*Eudistoma ovatum*, *E. reginum*, *E. tumidum* and *Polyclinum fungosum*) were encountered in the present study. The increasing number of new entries of ascidians and absence of previously reported species at this station may be due to changes in physico-geographical structures such as expanding of fishing harbour, establishment of new jetties and heavy traffic of fishing vessels.

The diversity of ascidians is influenced by the development of coastal patterns and environmental impacts (Carman et al. 2007). Ascidians are common inhabitants of harbours and marinas in both temperate and tropical waters (Monniot et al. 1991). They can easily be translocated by ships as well as boats and also through ballast water due to their motile larvae and sedentary adults. The dimension of ascidian diversity and increased invasive state of ascidians is influenced by coastal shipping patterns (Carman et al. 2011).

The Mandapam station has lots of substrata such as jetties, embedded rocks, small stones, fishing and coastguard vessels providing a suitable environment for the settlement of ascidians in a subtropical habitat.

Members of the family Polyclinidae occured in huge numbers throughout the study period with maximum colony size during summer season (March-May). *Ecteinascidia venui* was recorded in large numbers during premonsoon season (June-August). Ascidians are seasonal recruiters (Tamilselvi et al. 2012). Other ascidian species had sporadic occurrences throughout the study period.

## Supplementary Material

Supplementary material 1 Members of various ascidian families recorded in the present study Data type: Statistical
Brief description: Figure shows the number of genus and species represented in the families of ascidians.
File: oo_79760.xlsx Jaffarali, Akram & Arshan

XML Treatment for Ecteinascidia thurstoni

XML Treatment for Ecteinascidia turbinata

XML Treatment for Ecteinascidia venui

XML Treatment for Symplegma oceania

XML Treatment for Styela canopus

XML Treatment for Microcosmus exasperatus

XML Treatment for Microcosmus propinquus

XML Treatment for Eudistoma carnosum

XML Treatment for Eudistoma gilboviride

XML Treatment for Eudistoma pyriforme

XML Treatment for Eudistoma viride

XML Treatment for Synoicum galei

XML Treatment for Polyclinum glabrum

XML Treatment for Polyclinum nudum

XML Treatment for Polyclinum saturnium

XML Treatment for Polyclinum solum

XML Treatment for Polyclinum tenuatum

XML Treatment for Aplidiopsis confluata

XML Treatment for Trididemnum caelatum

XML Treatment for Trididemnum clinides

XML Treatment for Trididemnum cyclops

XML Treatment for Trididemnum savignii

XML Treatment for Trididemnum vermiforme

XML Treatment for Didemnum psammatodes

XML Treatment for Didemnum spadix

XML Treatment for Lissoclinum bistratum
